# Single-Item Happiness Measure Features Adequate Validity Among Adolescents

**DOI:** 10.3389/fpsyg.2022.884520

**Published:** 2022-06-28

**Authors:** Justė Lukoševičiūtė, Geneviève Gariepy, Judith Mabelis, Tania Gaspar, Roza Joffė-Luinienė, Kastytis Šmigelskas

**Affiliations:** ^1^Department of Health Psychology, Faculty of Public Health, Medical Academy, Lithuanian University of Health Sciences, Kaunas, Lithuania; ^2^Faculty of Public Health, Research Institute, Medical Academy, Lithuanian University of Health Sciences, Kaunas, Lithuania; ^3^Public Health Agency of Canada, Guelph, ON, Canada; ^4^Medical Research Council, Chief Scientist Office Social and Public Health Sciences Unit, Glasgow, United Kingdom; ^5^CLISSIS, Psychology and Educational Sciences Institute, Universidade Lusíada, Lisbon, Portugal

**Keywords:** happiness, validity, well-being, health complaints, bullying, violence, social support

## Abstract

**Background:**

Happiness is becoming increasingly relevant in recent research, including adolescents. Many studies are using the single-item measure for adolescent happiness, however, its validity is not well known. We aimed to examine the validity of this measure among adolescents in three countries from distinct European regions – Eastern (Lithuania), Southern (Portugal), and Western (Scotland).

**Materials and Methods:**

The analysis included data from Health Behaviour in School-aged Children (HBSC) study from three countries and three last surveys (2009/10, 2013/14, and 2017/18). The total sample comprised 47,439 schoolchildren. For validity, the indicators reflecting subjective health, life satisfaction, quality of life, well-being, social support, health complaints, bullying, and self-directed violence were assessed. The calculations were conducted in the total sample and by gender, age, survey year, and country.

**Results:**

The different indicators of concurrent and convergent validity revealed consistent correlations with happiness, with better well-being, health, and subjective perceptions being related to higher happiness. Meanwhile, health complaints, bullying behaviors, and self-directed violence were related to lower happiness. The subgroup differences were consistent across gender, age groups, countries, and survey rounds. The extent of differences was more expressed among girls.

**Conclusion:**

The single item for adolescent happiness measurement features a consistent pattern of validity concerning indicators of concurrent and convergent validity. Higher self-reported happiness is associated with better mental and physical health and well-being, and less expressed negative factors (complaints, bullying, and self-directed violence). In addition, among girls the correlations tend to be stronger than boys.

## Introduction

Adolescence is a time when young people face many challenges relating to their biological and psychological changes. It is the period from ages 10 to 19 years which is specific stage of human development laying the foundations of good health [World Health Organization [WHO], 2022]. Positive youth development could offer young people a way to acquire and strengthen resources to enable them to grow and flourish throughout life (Park, 2004). There has been an increase in the number of studies looking at positive psychology, but most happiness studies have been conducted in adults (Işik and Üzbe Atalay, 2019), even though happiness is associated with positive health and healthier development during adolescence (Steptoe, 2019).

Happiness is generally defined as a mental or emotional state of well-being, characterized by positive or pleasant emotions ranging from contentment to intense joy (Sundriyal and Kumar, 2014). According to Veenhoven (2010), happiness may also be defined as the appreciation of one’s life as a whole, involving both a cognitive and emotional evaluation of life (Veenhoven, 2008; Lucas and Diener, 2009).

The most widely used and easiest measure of adolescent happiness is a single item asking the adolescents to rate how happy they are. However, information about the validity of this question among adolescents is still scarce. A recent systematic review on adolescent happiness (Lukoševičiūtė et al., 2022) found that almost half of the studies have evaluated happiness using single-item measures. When young people were asked to define happiness, they mentioned not having negative thoughts or feelings, and experiencing positive feelings (Işik and Üzbe Atalay, 2019). Adolescents described a happy person as someone helpful, cheerful, optimistic, and expresses his or her positive feelings, and includes behavior such as helping others, laughing, having fun, and making people happy. Happiness also meant spending time with family and friends. Several studies have shown that academic success, satisfaction of needs, more leisure time, and having future plans are also important to what adolescents perceive to make someone happy (Işik and Üzbe Atalay, 2019).

According to the literature, different factors are linked with adolescent happiness, including subjective well-being and health, social support, experience of bullying, and self-directed violence. A growing body of research consistently demonstrates a close connection between health and happiness (Veenhoven, 2008; Diener and Chan, 2011; Ngamaba et al., 2017; Steptoe, 2019). Theories suggest that this relationship is bi-directional. Good health is an important predictor of happiness, and greater happiness is associated with good health through behavioral and biological processes (Steptoe, 2019). The robust health-happiness association has been shown in cross-national samples of adolescents. For instance, Van de Wetering et al. (2010) found that Dutch adolescents who reported better health were more likely to report greater happiness. Among Brazilian adolescents, Câmara and Strelhow (2019) also found that a higher level of current happiness was a significant predictor of better self-perceived health. Esteban-Gonzalo et al. (2020) similarly reported an association between higher levels of happiness and better self-rated health in Spanish adolescent girls and boys.

Lower subjective happiness further correlates with a range of physical and mental health symptoms in adolescents. A study among Spanish adolescents found that a lower level of happiness correlated with more physical and mental health complaints, including symptoms of somatization, depression, and anxiety (Garaigordobil, 2015). Freire and Ferreira (2020) found that depressive symptoms were negatively associated with happiness, while Shen et al. (2018) revealed that lower happiness was related to sleep problems in Australian adolescents. In sum, the studies with adolescents point to a consistent positive association between happiness and various measures of physical and mental health.

Additionally, social support is considered an important aspect of overall health and well-being. Adolescents receive social support from different groups (parents, wider family members, teachers, friends, and classmates), and they may influence adolescents in different ways (Tomás et al., 2020). Social support serves as a buffer for adolescents in stressful life events, with the perception of social support, rather than the actual support received, serving as a better predictor of a person’s ability to cope (Alshammari et al., 2021).

Ryan and Deci (2000) argue that close relationships with other people are a basic psychological need, with other researchers further stating that positive social relationships are a prerequisite for happiness (Diener and Oishi, 2005). A number of studies demonstrate the association between positive social relationships and subjective well-being in young people (Uusitalo-Malmivaara and Lehto, 2013). For instance, positive family relationships predict higher happiness in both genders (Uusitalo-Malmivaara and Lehto, 2013). Likewise, friendships are seen in children as both a source of happiness and, in case of conflict, a source of unhappiness (Holder and Coleman, 2015).

In addition to the family, because adolescents spend a significant amount of time at school, the school environment can impact their well-being (O’Reilly et al., 2018). Teacher support is not only considered necessary for academic development but also may impact students’ emotional outcomes (Lei et al., 2018), while better relationships with school staff are also associated with positive subjective well-being (Moore et al., 2018).

Adolescent happiness is also associated with experiencing less violence or conflict, such as quarreling and bullying (López-Pérez and Zuffianò, 2020). Uusitalo-Malmivaara and Lehto (2016) showed that bullying victims reported lower levels of well-being and happiness, with adolescents who experienced dual victimization (bullying and cyberbullying) reporting even more negative impact on their happiness and depression. Moreover, experiencing bullying and cyberbullying is negatively correlated with indicators of subjective well-being such as optimism, global and school-related happiness, and specific domains of life satisfaction (Navarro et al., 2015).

Finally, evidence suggests that adolescents reporting a lower level of happiness were at greater risk of deliberate self-harm (Lung et al., 2020). The latter includes any form of intentional self-injury, irrespective of motive or suicidal intent (Curtis et al., 2018). Self-harm is a relevant phenomenon among adolescents and includes various types of expressions of injury on the body (Remaschi et al., 2015). Low levels of happiness correlate with prolonged stress and mental health difficulties (Wersebe et al., 2018) which in turn are highly associated with self-directed violence among adolescents, including cases with suicidal intentions (Abi-Jaoude et al., 2020). Studies examining the relationship of distress and mental health problems to self-directed violence show that they are associated, although these studies rarely include happiness (Kidger et al., 2015; Curtis et al., 2018).

This review of the literature suggests that the perception of happiness among adolescents is a relevant construct when assessing their well-being and health-related behaviors and outcomes. Given the associations between happiness and other important aspects outlined above, measuring adolescent happiness may provide not just a measure of construct but also an important insight into the general well-being of young people. This information could be used to inform the impact and need for public health interventions and policies. A single-item measure of happiness could readily be added to surveys to help monitor and study adolescent happiness, but its validity among adolescents is unclear, including cross-national differences in the validity due to language interpretation, culture, age, and gender differences across adolescence. Using survey data from the international Health Behaviour in School-aged Children (HBSC) survey, we, therefore, aimed to examine the validity of a single-item happiness measure among adolescents in three countries from distinct European regions – Eastern (Lithuania), Southern (Portugal), and Western (Scotland) – using psychological, health, and social variables that are known to vary with happiness and explore possible gender, age, country, and temporal differences across surveys.

## Materials and Methods

### Study Procedure

The Health Behavior in School-aged Children survey is a cross-sectional survey currently conducted in 50 countries across Europe and North America in collaboration with the World Health Organization. The data were collected using standardized protocols [2009/10 (Currie et al., 2010), 2013/14 (Currie et al., 2014), and 2017/18 (Inchley et al., 2018)] and provides nationally representative samples of 11-, 13-, and 15-year-old boys and girls for each participating country, using a cluster sampling method (with the school classes as the primary sampling unit) and ensuring representation by age, gender, and school type. For this study, we included data from Lithuania, Portugal, and Scotland as these participating countries had included the single-item measure of happiness in their surveys during different years and represent a culturally diverse sample of adolescents from different regions of Europe. We included data from the three most recent survey rounds (2009/10; 2013/14; 2017/18), which provide information on the relative stability of the happiness measure over time and increase the sample size as well as study power.

Each country applied for ethical approval of the study in their own country. In Lithuania and Scotland, data were collected through a school-based survey using paper-pencil questionnaires, while in Portugal data were collected using paper-pencil questionnaires in 2010 and electronic questionnaires in 2014 and 2018.

### Participants

This analysis involved the study sample of 47,439 schoolchildren (mean age 13.7 ± 1.71 years). It was designed to be a representative sample of schoolchildren in Lithuania, Portugal, and Scotland. Detailed characteristics of the study sample by country and survey round are presented in Table 1.

**TABLE 1:** Main characteristics of study sample size by country and survey round.

Gender and age	Country						Round					
	Lithuania		Portugal		Scotland		2009/10		2013/14		2017/18	
Boys 11–12 years	2,019	17.6%	2,735	15.7%	2,796	16.1%	2,697	15.6%	2,820	16.2%	2,033	17.6%
Boys 13–14 years	1,974	17.2%	3,181	18.3%	2,912	16.8%	2,812	16.3%	3,224	18.6%	2,031	17.5%
Boys 15–16 years	1,903	16.6%	2,373	13.7%	2,828	16.3%	3,053	17.7%	2,551	14.7%	1,500	13.0%
Girls 11–12 years	1,970	17.2%	2,904	16.7%	2,954	17.0%	2,706	15.7%	2,962	17.0%	2,160	18.7%
Girls 13–14 years	1,975	17.2%	3,342	19.2%	2,936	16.9%	2,806	16.2%	3,253	18.7%	2,194	19.0%
Girls 15–16 years	1,641	14.3%	2,832	16.3%	2,953	17.0%	3,200	18.5%	2,569	14.8%	1,657	14.3%
Total	11,482	100.0%	17,367	100.0%	17,379	100.0%	17,274	100.0%	17,379	100.0%	11,575	100.0%

### Measures

**Happiness** was measured using a single item: “*In general, how do you feel about your life at present?*. “The responses ranged from *“I feel very happy”* to *“I am not happy at all”* and later reversed to make higher scores indicating higher level of happiness.

**Self-rated health** was measured with a single item asking, *“Would you say your health is*…*?”* with four response categories (*“excellent,” “good,” “fair,” and “poor”).* Later the scores were reversed to make higher scores indicating better health.

**The Multiple Health Complaints** scale asks about the frequency of eight common health symptoms in the past 6 months: headache, stomachache, backache, sleeping difficulties, feeling low, irritability or bad mood, feeling nervous, and dizziness. Higher scores indicated higher frequency of symptoms. All eight health complaints were also combined and coded into two groups of “two or more health complaints more than once a week” and “less than that” (Haugland and Wold, 2001).

**Social support.** We included perceived levels of social support that adolescents feel from four different people’s groups:

• **Teacher support.** Three questions ask whether the adolescents feel accepted by their teachers, trust their teachers, and feel that their teachers cared about them. The items are based on the Teacher and Classmate Support scale (Torsheim et al., 2000). Responses are on a 5-point scale from 1 (*“strongly agree”*) to 5 (*“strongly disagree”*). The overall mean score was calculated as the mean score of items, with lower scores indicating a perception of higher support. Further the scores were reversed to make higher scores indicating higher level of teacher support.

• **Student support.** Three questions ask whether the adolescent enjoys being with other students, feels accepted by them, and if the students are kind and helpful. The items are based on the Teacher and Classmate Support scale (Torsheim et al., 2000). The five response options range from 1 (*“strongly agree”*) to 5 (*“strongly disagree”*). The reversed mean score was calculated with higher scores indicating a perception of higher support.

• **Friend support.** Four questions from the Multidimensional Scale of Perceived Social Support (MSPSS) ask about the support received from friends, including if their friends help them, are they able to rely on friends, and discuss problems. There are seven response options ranging from 1 (*“very strongly disagree”*) to 7 (*“very strongly agree”*) (Zimet et al., 1988). The responses were combined and a mean score was calculated, with higher scores indicating a perception of higher support.

• **Family support.** Family support was measured using the family aspects of MSPSS (Zimet et al., 1988). Adolescents were asked if they feel their family tries to help them, if they get emotional support from their family when needed, if they can talk to their family about problems, and if their family helps them make decisions. The responses are scored on a 7-point Likert scale, ranging from 1 (*“very strongly disagree”*) to 7 (*“very strongly agree”*). Higher mean scores indicate a stronger perception of social support.

**Life satisfaction** was measured using the Cantril ladder (Cantril, 1965). The top of the ladder (10) indicates *“the best possible life”* and the bottom (0) means *“the worst possible life.”*

The Kidscreen-10 Index (Ravens-Sieberer, 2006) was used to evaluate **health-related quality of life**. It is unidimensional index with 10 items ranging from 1 (*“never”*) to 5 (*“always”*). The overall mean score of the 10 items was used in the analysis. Higher scores indicate better quality of life.

Schoolchildren’s **well-being** was assessed using the *WHO-5 Well-being Index* (World Health Organization, 1998). Five statements ask about the frequency of positive feelings over the past 2 weeks with responses on a scale from 0 (*“at no time”*) to 5 (*“all the time”*). The overall mean score was used in this analysis with higher scores indicating higher level of well-being.

**Bullying and cyberbullying** included both victims and perpetrators of bullying or cyberbullying. Reports of being bullied at least 2 or 3 times a month in the past 2 months were considered to be chronic victims and perpetrators of bullying or cyberbullying (Harel-Fisch et al., 2011; Johnston et al., 2015). Higher scores indicate more frequent bullying.

**Self-directed violence** included suicidal ideation (whether the children have considered, planned, or attempted suicide) and self-harming behavior in the last 12 months. These variables had responses *“yes”* or *“no.”*

The sociodemographic **indicators** included in this study were age, gender, country, and year of data collection.

The internal consistency of the scales was analyzed by gender and age group using Cronbach’s alpha. The calculations showed consistent results by age and gender; differences between these groups were mainly below 0.02 and at most 0.06. In contrast, the consistency by country and survey year revealed larger variation, although in the majority of cases the alphas were high or very high (Table 2).

**TABLE 2:** Internal consistency of the scales by country and survey round.

Variable	Country			Round		
	Lithuania	Portugal	Scotland	2009/10	2013/14	2017/18
Kidscreen-10 Index	n.a.	0.74	0.83	0.80	0.60	0.83
WHO-5 Index	n.a.	n.a.	0.86	n.a.	n.a.	0.86
Family support	n.a.	0.90	0.95	0.92	0.89	0.96
Teacher support	0.84	0.84	0.87	0.86	0.85	0.86
Student support	0.79	0.75	0.78	0.76	0.80	0.77
Friend support	0.92	0.93	0.95	0.92	0.93	0.94

*n.a., not available.*

### Statistical Analysis

The data were analyzed using IBM SPSS Statistics, version 27 (IBM Corp, 2020). The descriptive analysis included the calculation of the means with standard deviations (±SD) and percentages of health behaviors and perceptions. The items were dichotomized based on the cut-offs used in the international HBSC study protocols and outlined in the “Measures” subsection.

The validity of the happiness item was examined in relation to other indicators as criterion concurrent validity (life satisfaction, health-related quality of life, well-being, and social support measures) and construct convergent validity (subjective health, physical and psychological complaints, bullying, and self-directed violence). The indicators for validity were chosen based on the previous literature on happiness. The associations were calculated using a Spearman’s rank correlation (rho) for ordinal indicators and point-biserial correlation (rpb) for dichotomous indicators of suicidal ideation. The strength of associations was expressed by looking at differences between happy and unhappy adolescents. To assess the consistency of associations, the calculations were conducted not only for the whole sample but also by gender, age, survey year, and country.

The statistical significance level was set at *p* < 0.05.

## Results

### Descriptive Statistics

**FIGURE 1:**
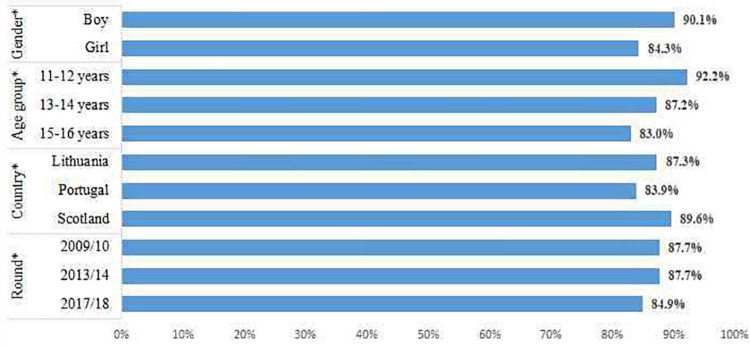
Prevalence of happy adolescents by gender, age group, country, and survey round.

In the overall sample, the majority of adolescents (87.1%) reported feeling happy (quite happy or very happy) (Figure 1). Lower levels of happiness were observed amongst girls and older adolescents, in Portugal, and the most recent survey round of 2017/18.

The descriptive analysis of indicators used for validity revealed some differences by country and survey round (Table 3). However, none of the countries or survey years were associated with consistently better or worse outcomes. This suggests a good basis for comparability of validation measures across countries and survey years. Note that some of the variables were not collected in every country at every round, though the majority were collected in all three countries in all years. The cyberbullying measure was introduced in 2013/14 and the well-being measure – in 2017/18.

**TABLE 3:** Description of validity indicators by country and survey round.

Variable	Round			Country		
	2009/10	2013/14	2017/18	Lithuania	Portugal	Scotland
Life satisfaction (lowest = 0, highest = 10)	7.5 ± 1.92	7.7 ± 1.93	7.7 ± 1.85	7.7 ± 2.03	7.5 ± 1.87	7.7 ± 1.86
Quality of life (lowest = 10, highest = 50)	38.4 ± 6.25	33.6 ± 5.35	36.7 ± 7.52	n.a.	36.2 ± 6.60	37.0 ± 6.49
Well-being (lowest = 1, highest = 6)	n.a.	n.a.	3.9 ± 1.14	n.a.	n.a.	3.9 ± 1.14
Family support (lowest = 1, highest = 7)	5.8 ± 1.41	5.6 ± 1.63	5.7 ± 1.81	n.a.	5.9 ± 1.52	5.4 ± 1.85
Teacher support (lowest = 1, highest = 5)	3.7 ± 0.87	3.8 ± 0.91	3.8 ± 0.87	3.8 ± 0.89	3.8 ± 0.85	3.8 ± 0.92
Student support (lowest = 1, highest = 5)	3.9 ± 0.80	3.8 ± 0.84	3.8 ± 0.80	3.7 ± 0.89	4.0 ± 0.76	3.7 ± 0.78
Friend support (lowest = 1, highest = 7)	5.9 ± 1.39	5.4 ± 1.73	5.3 ± 1.82	5.4 ± 1.79	5.6 ± 1.61	5.2 ± 1.83
Health (excellent or good)	83.3%	85.8%	85.5%	86.5%	87.2%	81.2%
Health complaints (not more than 1 from 8 once a week)	70.5%	71.9%	66.9%	66.5%	73.2%	69.3%
Headache (less than once a week)	83.4%	84.4%	85.9%	81.3%	86.3%	84.5%
Stomachache (less than once a week)	90.5%	90.9%	93.4%	87.4%	94.4%	91.0%
Backache (less than once a week)	88.3%	88.8%	86.2%	88.3%	85.4%	90.4%
Feeling low (less than once a week)	84.2%	84.4%	81.3%	80.3%	85.1%	84.0%
Irritable (less than once a week)	78.8%	79.7%	76.2%	75.9%	81.7%	76.9%
Nervous (less than once a week)	81.3%	79.6%	74.9%	77.1%	78.8%	80.5%
Sleep difficulty (less than once a week)	80.0%	81.5%	75.7%	81.5%	82.6%	74.9%
Dizzy (less than once a week)	90.3%	90.0%	90.5%	88.0%	93.5%	88.4%
Bullied others (less than once or twice)	85.6%	60.6%	97.6%	77.9%	65.2%	94.6%
Been bullied (less than once or twice)	86.6%	82.8%	90.3%	72.3%	92.1%	89.5%
Cyberbullied others (less than once or twice)	91.7%	86.4%	98.4%	n.a.	92.6%	98.5%
Been cyberbullied (less than once or twice)	n.a.	94.5%	96.6%	n.a.	96.2%	95.4%
Considered suicide (no)	83.9%	76.1%	n.a.	78.8%	n.a.	n.a.
Planned suicide (no)	90.5%	86.2%	n.a.	87.7%	n.a.	n.a.
Attempted suicide (no)	92.9%	86.8%	n.a.	89.0%	n.a.	n.a.
Self-harm (no)	84.4%	79.7%	80.4%	n.a.	81.4%	n.a.

*n.a., not available.*

### Concurrent Validity

Concurrent validity was assessed using indicators of previously validated life satisfaction, well-being, and social support measures (Table 4). In total sample, happiness most strongly correlated with well-being (rho = 0.62), life satisfaction (rho = 0.56), and quality of life (rho = 0.42). Likewise, the different social support also significantly correlated with happiness but to smaller extent. The subgroup analysis demonstrated that correlations were consistent and similar across gender, age groups, countries, and survey rounds. The differences in correlations varied a little, with slightly stronger correlations observed among girls and adolescents from Scotland. All observed correlations were highly significant (rho < 0.001).

**TABLE 4:** Concurrent validity of happiness in relation to life satisfaction, well-being, and social support: Spearman’s correlation.

Variable	Total sample*	Gender*		Age group*			Round*			Country*		
		Boys	Girls	11–12 years	13–14 years	15–16 years	2009/10	2013/14	2017/18	Lithuania	Portugal	Scotland
Life satisfaction (min = 0, max = 10)	0.56	0.52	0.60	0.53	0.54	0.56	0.54	0.59	0.57	0.59	0.48	0.61
Quality of life (min = 10, max = 50)	0.42	0.36	0.47	0.34	0.37	0.45	0.45	0.37	0.49	n.a.	0.33	0.58
Well-being (min = 1, max = 6)	0.62	0.58	0.65	0.53	0.64	0.60	n.a.	n.a.	0.62	n.a.	n.a.	0.62
Family support (min = 1, max = 7)	0.32	0.28	0.37	0.28	0.33	0.31	0.34	0.35	0.30	n.a.	0.34	0.34
Teacher support (min = 1, max = 5)	0.31	0.28	0.34	0.27	0.27	0.25	0.28	0.30	0.36	0.32	0.29	0.32
Classmate support (min = 1, max = 5)	0.28	0.27	0.28	0.31	0.26	0.22	0.24	0.30	0.32	0.29	0.24	0.37
Friend support (min = 1, max = 7)	0.18	0.20	0.21	0.20	0.19	0.16	0.20	0.19	0.18	0.17	0.21	0.20

n.a., not available, *all p < 0.001.

### Convergent Validity

Convergent validity of the happiness item was assessed using measures of self-reported health, health complaints, bullying behaviors and perceptions, and self-directed violence (Table 5). Overall, the correlations tended to be in general lower than in case of concurrent validity indicators. In total sample, it most strongly correlated with total amount of frequent symptoms (rho = −0.34), especially with feeling low (rho = −0.40), irritability or bad temper (rho = −0.31), and feeling nervous (rho = −0.31). The close-to-moderate correlations were also observed with general perception of health (rho = 0.31), slightly lower – with suicidal and self-harming indicators, especially considering suicide (rpb = −0.26). Other indicators, including somatic symptoms and bullying behaviors and perceptions, were weakly though significantly correlated with happiness of adolescents.

**TABLE 5:** Convergent validity of happiness in relation to subjective health, health complaints, bullying behaviors and perceptions, and self-directed violence: Spearman’s correlation.

Variable	Total sample *	Gender *		Age group *			Round *			Countries *		
		Boys	Girls	11–12 years	13–14 years	15–16 years	2009/10	2013/14	2017/18	Lithuania	Portugal	Scotland
Health	0.31	0.27	0.32	0.28	0.30	0.27	0.26	0.35	0.31	0.34	0.31	0.34
Total frequent symptoms	−0.34	−0.25	−0.39	−0.28	−0.34	−0.34	−0.29	−0.34	−0.42	−0.29	−0.30	−0.41
Headache	−0.21	−0.14	−0.25	−0.16	−0.21	−0.20	−0.18	−0.23	−0.25	−0.19	−0.22	−0.26
Stomach ache	−0.17	−0.12	−0.18	−0.14	−0.17	−0.15	−0.14	−0.19	−0.17	−0.17	−0.16	−0.23
Back ache	−0.20	−0.16	−0.22	−0.16	−0.17	−0.16	−0.17	−0.22	−0.21	−0.15	−0.19	−0.22
Feeling low	−0.40	−0.31	−0.46	−0.30	−0.40	−0.43	−0.35	−0.40	−0.47	−0.33	−0.40	−0.46
Irritability or bad temper	−0.31	−0.25	−0.36	−0.25	−0.30	−0.31	−0.27	−0.33	−0.37	−0.31	−0.31	−0.35
Feeling nervous	−0.31	−0.25	−0.34	−0.25	−0.30	−0.32	−0.28	−0.34	−0.33	−0.31	−0.28	−0.34
Difficulties in sleeping	−0.25	−0.20	−0.27	−0.21	−0.26	−0.24	−0.20	−0.27	−0.28	−0.22	−0.23	−0.32
Feeling dizzy	−0.19	−0.14	−0.22	−0.15	−0.21	−0.17	−0.14	−0.21	−0.23	−0.19	−0.17	−0.26
Bullying others	−0.10	−0.12	−0.10	−0.14	−0.11	−0.08	−0.18	−0.05	−0.08	−0.11	−0.07	−0.12
Been bullied past months	−0.16	−0.17	−0.17	−0.21	−0.20	−0.14	−0.16	−0.17	−0.16	−0.20	−0.08	−0.18
Cyber bullied others	−0.11	−0.11	−0.13	−0.12	−0.13	−0.10	−0.10	−0.17	−0.10	n.a.	−0.10	−0.12
Been cyber bullied	−0.13	−0.10	−0.14	−0.11	−0.13	−0.15	n.a.	−0.09	−0.15	n.a.	−0.11	−0.18
Considered suicide **	−0.26	−0.16	−0.33	n.a.	−0.28	−0.27	−0.25	−0.29	n.a.	−0.26	n.a.	n.a.
Planned suicide **	−0.19	−0.10	−0.26	n.a.	−0.22	−0.18	−0.15	−0.22	n.a.	−0.19	n.a.	n.a.
Attempted suicide **	−0.17	−0.10	−0.24	n.a.	−0.21	−0.17	−0.12	−0.21	n.a.	−0.17	n.a.	n.a.
Self−harm behaviour **	−0.22	−0.13	−0.28	n.a.	−0.22	−0.23	−0.17	−0.26	−0.22	n.a.	−0.22	n.a.

n.a., not available, *all p < 0.001, **point-biserial correlation.

Similarly like in case of concurrent validity indicators, the convergent validity measures correlated stronger with happiness among girls and Scotland’s adolescents. No consistent pattern was observed based on schoolchildren’s age group and survey year.

## Discussion

Adolescent happiness is an important construct that relates to a range of positive health and behavioral outcomes and the overall well-being of young people. We aimed to assess the validity of a single-item happiness measure, overall and across gender, age groups, survey years, and countries, using a range of behavioral, physical, psychological, and social factors shown to be relevant to happiness. Our findings showed that all the selected measures were consistently associated with the single-item measure of happiness. This was observed with convergent and concurrent validity measures. The results indicate that a single-item happiness measure is a valid tool for population-based adolescent studies, yielding comparable results across gender, age, countries, and time. We discuss the findings from a perspective of the related constructs.

Adolescent self-reported happiness was strongly associated with measures of both mental and physical health, providing evidence for the validity of the single happiness item. In line with previous work, we observed marked differences between those who reported being happy and unhappy in self-rated health (Van de Wetering et al., 2010; Câmara and Strelhow, 2019; Esteban-Gonzalo et al., 2020), physical health complaints (Garaigordobil, 2015; Shen et al., 2018), and psychological health complaints (Freire and Ferreira, 2020). In addition, our results show that the total number of health complaints also correlated with happiness. Overall, self-reported happiness correlated most strongly with psychological health complaints, especially feeling low. This is unsurprising given the close inverse relationship between happiness and poor mental health. Although theoretically distinct concepts, unhappiness may be interpreted as a symptom of poor mental health and vice versa. Moreover, mood states at the time of the survey, such as feeling low, may negatively influence the report of overall happiness. Across all health measures, the associations with happiness were stronger among girls than boys, indicating that girls’ assessment of happiness may be more influenced by their physical and mental health than among boys.

This study supports previous theories and evidence that social support is associated with happiness and that support from different sources is related to adolescent happiness. Whilst it is beyond the scope of this manuscript to compare the relative importance of each form of social support, we see there is particular importance on family support compared with friend support, especially amongst girls. This reflects previous research which indicates that family support impacts more on girls’ happiness (Uusitalo-Malmivaara and Lehto, 2013). Contrary to some of the literature (Holder and Coleman, 2015), we do not see the importance of family relations decreasing with age.

Within this study, it was not possible to establish the direction of association between happiness and social support – so, this is likely to be bi-directional. Young persons who are unhappy may perceive their relationships in a more negative way (Natvig et al., 2003). In addition, not having supportive relationships may be a source of unhappiness and unhappy people may struggle to establish good relationships due to their maladjusted behavior (Uusitalo-Malmivaara and Lehto, 2013). It may also be argued that social support is indirectly associated with happiness. For example, it is through social support that young people develop important attributes such as self-concept, self-efficacy, self-esteem, and school engagement which themselves may bring about greater happiness (Ikiz and Savi, 2010; Holder and Coleman, 2015; Tomás et al., 2020).

Social contacts at school play an important part in adolescents’ lives. There may therefore be merit in teaching young people the skills to establish and maintain good social relationships in the school environment (Natvig et al., 2003; Tomás et al., 2020) with schools being not only places of learning but also a social institution where adolescents develop their social functioning (Bond et al., 2004). Moreover, for young people from less supportive families, fostering stronger social relationships, and support outside of home may be a way to improve happiness (Uusitalo-Malmivaara and Lehto, 2013).

Concerning bullying behaviors (victimization and perpetration), we found that being a victim was more slightly stronger related to happiness than being a bully, with a greater effect through face-to-face bullying than cyberbullying. Several authors (Navarro et al., 2015; Fullchange and Furlong, 2016; Uusitalo-Malmivaara and Lehto, 2016) concluded that the impact of bullying and cyberbullying on adolescents is far-reaching and can have medium- and long-term consequences for young people’s development. Kwan et al. (2020) argue that bullying and cyberbullying can be a consequence or a source of mental health problems and unhappiness in children and adolescents. This aspect is even more significant when the same adolescent is both victim and perpetrator. Nonetheless, in our study the correlation between bullying and happiness was relatively low.

Our study demonstrated that happiness among adolescents correlates negatively with self-directed violence. This reflects results found in other studies that examined the associations between subjective happiness and self-directed violence: a recent study in Taiwan found that adolescents perceiving a lower level of happiness are at higher risk of deliberate self-harm (Lung et al., 2020). Several studies about suicidal ideation also showed that youth with suicidal ideation reported lower levels of subjective happiness than those without suicidal ideation (Choi et al., 2014; Kim, 2017; Shin and Lee, 2020).

### Limitations

We should acknowledge that our study also had some limitations. While our study investigated the validity of single-item happiness measure, we did not assess its reliability in a test-retest manner. Nonetheless, we consider our study to be of high scientific and practical value, as it included a large sample, a decade frame of measurements, and a relatively diverse sample from three culturally different countries Europe amongst a population from early to mid-adolescence. On the other hand, we compared the cross-national validity of the happiness item across three European countries, so the results may not be generalizable to other countries or cultures (moreover, some indicators in some countries were not available in some survey rounds). Also, since suicidal and self-harm indicators were less systematically collected across the analyzed countries, the differences between survey rounds should be interpreted with caution.

## Conclusion

The single item for adolescent happiness measurement features a consistent pattern of validity in relation to indicators of concurrent and convergent validity. Specifically, higher self-reported happiness is associated with better mental and physical health and well-being. Stronger sense of happiness correlates with higher self-rated health, quality of life, and social support as well as lower levels of physical and psychological complaints, bullying, and self-directed violence. In addition, these correlations among girls were slightly stronger than boys.

## Data Availability Statement

The raw data supporting the conclusions of this article will be made available by the authors, without undue reservation.

## Ethics Statement

The studies involving human participants were reviewed and approved by institutional ethics bodies in Lithuania (Kaunas Regional Biomedical Research Ethics Committee), Portugal (Health Ethics Committee of the Centro Hospitalar de São João), and Scotland (University of St Andrews’ University and Teaching Research Ethics Committee). In Lithuania written informed consent to participate in this study was provided by the participants of the study and their parents or legal guardians. In Portugal and Scotland, written informed consent from the participants and legal guardian was not required to participate in this study in accordance with the national legislation and the institutional requirements.

## Author Contributions

JL and KŠ framed the overall concept of study, conducted the analyses, and had overall input to all parts of the manuscript. GG, JM, TG, and RJ-L had main inputs in introduction and discussion, but also essentially revised all other parts of manuscript. All authors contributed to the article and approved the submitted version.

## Conflict of Interest

The authors declare that the research was conducted in the absence of any commercial or financial relationships that could be construed as a potential conflict of interest.

## Publisher’s Note

All claims expressed in this article are solely those of the authors and do not necessarily represent those of their affiliated organizations, or those of the publisher, the editors and the reviewers. Any product that may be evaluated in this article, or claim that may be made by its manufacturer, is not guaranteed or endorsed by the publisher.
